# Dr. Theophilus Parvin

**Published:** 1881-11-20

**Authors:** 


*Dr. Theophilus Parvin* has been elected to the Chair of Obstetrics and Gynæcology, in the Louisville University. A good appointment.

☞President Cabell, of National Board of Health, in his report to the Secretary of the Treasury, complains of the failure of Congress to appropriate moneys sufficient to enable the Board to prosecute the many investigations which have been instituted, some of which are of the highest importance.

